# A novel SATB1 binding site in the BCL2 promoter region possesses transcriptional regulatory function^☆^

**DOI:** 10.1016/S1674-8301(10)60060-7

**Published:** 2010-11

**Authors:** Feiran Gong, Luan Sun, Yujie Sun

**Affiliations:** a Key Laboratory of Human Functional Genomics of Jiangsu Province,; b Department of Cell Biology,; c Cancer Center, Nanjing Medical University, Nanjing, Jiangsu 210029, China

**Keywords:** BCL2, promoter, special AT-rich sequence-binding protein 1, transcriptional regulation

## Abstract

BCL2 is a key regulator of apoptosis. Our previous work has demonstrated that special AT-rich sequence-binding protein 1 (SATB1) is positively correlated with BCL2 expression. In the present study, we report a new SATB1 binding site located between P1 and P2 promoters of the *BCL2* gene. The candidate SATB1 binding sequence predicted by bioinformatic analysis was investigated *in vitro* and *in vivo* by electrophoretic gel mobility shift assays (EMSA) and chromatin immunoprecipitation (ChIP). One 25-bp sequence, named SB1, was confirmed to be SATB1 binding site. The regulatory function of SB1 and its relevance to SATB1 were further examed with dual-luciferase reporter assay system in Jurkat cells. We found that SB1 could negatively regulate reporter gene activity. Mutation of SATB1 binding site further repressed the activity. Knockdown of SATB1 also enhanced this negative effect of SB1. Our data indicate that the SB1 sequence possesses negative transcriptional regulatory function and this function can be antagonized by SATB1.

## Introduction

*BCL2*, originally identified in B-cell lymphoma as a proto-oncogene, is not only a key regulator of apoptosis[1], but also involved in DNA repair, cell cycle and differentiation control[2]–[5]. Given its fundamental importance for the cellular fate, BCL2 expression is finely tuned by a variety of environmental and endogenous stimuli and regulated at both the transcriptional and post-transcriptional levels[6]–[12]. At the transcriptional level, the expression of the *BCL2* gene is regulated by both positive and negative elements located within the promoter, coding regions and 3′-UTR[13]–[18]. *BCL2* has two promoters, P1 and P2. P1 is located 1,386 to 1,423 bp upstream of the translation start site, and is the major transcriptional promoter while P2, located 1.3 kb downstream from P1, has primary functions only in specific tissues, such as t (14;18) lymphoma cells and neuronal cells[19],[20]. Our previous investigation demonstrated that special AT-rich sequence-binding protein 1 (SATB1) positively regulated *BCL2* gene expression, and reduction of SATB1 expression resulted in decreased *BCL2* expression in Jurkat cells[13].

SATB1 is a matrix attachment region (MAR)-binding protein (MBP). It is expressed predominantly in thymocytes at high levels[21]. SATB1 belongs to a class of transcriptional regulators that function as a scaffold for several chromatin remodeling enzymes and hence regulates large chromatin domains[22]. During development and tumor progression, SATB1 regulates temporal and spatial expression of multiple genes[23].

To explore the regulatory role of SATB1 in *BCL2* gene transcription, we identified one SATB1 binding site (designated as SB1) located between P1 and P2 with electrophoretic gel mobility shift assay (EMSA) and chromatin immunoprecipitation (ChIP) based on the bioinformatic analysis. The regulatory function of SB1 and its relevance to SATB1 were examined with dual-luciferase reporter assay system. We found that SB1 could negatively regulate reporter gene activity. The negative effect of SB1 on the reporter gene activity could be antagonized by knockdown of SATB1 or mutation within the SATB1 binding site. Our data suggest that the SB1 sequence possesses negative transcriptional regulatory function and this function could be antagonized by SATB1.

## MATERIALS AND METHODS

### Cell lines and cell culture

Human T lymphoid cell line Jurkat was a generous gift from Dr. Krontiris' Laboratory at City of Hope National Medical Center in Los Angeles, USA. Jurkat cells were grown in RPMI 1640 medium supplemented with 10% FBS, 10 mmol/L HEPES, 100 U/mL penicillin and 10 µg/mL streptomycin. The cells were incubated at 37°C in a humidified atmosphere containing 95% air and 5% CO_2_.

### Nuclear extracts and electrophoretic mobility shift assays (EMSA)

Nuclear extracts were prepared using NE-PER nuclear and cytoplasmic extraction reagents (Pierce, USA) following the manufacturer's instructions. Jurkat cells were washed twice with phosphate-buffered saline, and then were centrifuged at 500 *g* for 3 min, and the pellet was suspended in cytoplasmic extraction reagent I and cytoplasmic extraction reagent II. After centrifugation at 15,000 *g* for 5 min, the pellet was treated with nuclear extraction reagent with vortexing for 15 sec every 10 min for a total of 40 min. After centrifugation at 15,000 *g* for 10 min, the supernatant was collected as the nuclear extract. The protein concentrations were measured using a Bio-Rad protein assay.

EMSA was performed using a gel shift assay kit following the manufacturer's instructions (Promega, USA). In brief, 10 µg of Jurkat nuclear extracts were incubated for 10 min at room temperature with gel shift binding buffer in the presence or absence of unlabeled probe before the addition of ^32^P-labeled probe. The sequences of the probes were as follows: SB1-F, 5′-CGAAAGGAATTGGAATAAAAATTTC-3′ and SB1-R, 5′-GAAATTTTTATTCCAATTCCTTTCG-3′. After a 20-min incubation at room temperature, the samples were resolved on a 5% polyacrylamide gel. For antibody-mediated supershift assay, reaction mixtures with antibody were incubated at room temperature for another 40 min before electrophoresis. Signals were recorded on X-ray film.

### Chromatin immunoprecipitation assay

ChIP assays were performed using the ChIP assay kit essentially as described by the manufacturer (Upstate, USA). Briefly, Jurkat cells (1×10^7^) were fixed in 1% formaldehyde for 10 min at room temperature. After cell lysis, genomic DNA was sheared into 200-1000 bp fragments using Sonics VCX130 (SONICS, USA). Sheared chromain was incubated with anti-SATB1 antibody or IgG overnight at 4°C. NaCl was added to the ChIP samples for 4 h at 65°C to reverse the cross-links. To purify the immunoprecipitated DNA, RNase and proteinase K were added, followed by phenol-chloroform extraction, ethanol precipitation and resuspension of the DNA in distilled water. The immunoprecipitated DNA was then amplified by PCR using primers corresponding to SB1 of BCL2. The primers used were synthesized: ChIP-F, 5′-ACCTTTCAGCATCACAGA-3′ and ChIP-R, 5′-AATCACGCGGAACACTTG-3′. The PCR cycling parameters were as follows: 30 sec at 95°C, 30 sec at 56°C, and 30 sec at 72°C, for 32 cycles. An aliquot of input genomic DNA was amplified by PCR along with aliquots of immunoprecipitated DNA to assess the relative binding of SATB1. The PCR products were subjected to gel electrophoresis, stained with ethidium bromide, and analyzed using the Molecular Imager Gel Doc XR System (Bio-Rad, USA).

### Construction of plasmids

Luciferase reporter construct containing SB1 was prepared using pGL3-promoter vector. The sequences (including the sites of restriction enzymes) were as follows: pGL3-F, 5′-CCGAAAGGAATTGGAATAAAAATTTCC-3′ and pGL3-R, 5′-TCGAGGAAATTTTTATTCCAATTCCTTTCGGAGCT-3′. The pGL3-promoter vectors were digested with the corresponding restriction enzymes (*Xho* I and *Sac*I, NEB, UK) and then used to construct the recombinant plasmids. The AT site was mutated to GC in the -217--193 construct using the QuikChange^®^ Site-Directed Mutagenesis Kit (Stratagene, USA). The primers used for mutagenesis are as follows with the SB1 sequence underlined and the mutated bases in boldface: mut-1, 5′-CCGAGCTCCGAAAGGA**GC**TGGAATAAAAATTTCC-3′; mut-2, 5′-CCGAAAGGAATTGGA**GC**AAAAATTTCCTCGAG-3′; mut-3, 5′-GGAATTGGAATAAAA**GC**TTCCTCGAGATCTGCG-3′. All plasmid sequences were confirmed by sequencing.

SATB1-specific siRNA sequences were synthesized according to those as reported by Han *et al.*[24] and inserted into the pGCsi-H1/Neo/GFP/siNEGative vector (Genscript, USA), which coexpresses GFP to allow identification of transfection efficiency. The SATB1 shRNA sequence was: SATB1-shRNA 5′-GTCCACCTTGTCTTCTCTC-3′. The non-specific shRNA sequence was: control-shRNA 5′-ACGTGACACGTTCGGAGAA-3′[13]. All constructs were confirmed by sequencing.

### Transient transfection and luciferase assays

Jurkat cells were transfected with 20 µg luciferase reporter plasmids plus 10 ng pRL vectors using an electroporator (Bio-Rad, USA) at 975 µF and 250 V in a 0.4 cm cuvette at a concentration of 2×10^7^ cells/350 µL in RPMI 1640 medium containing 10% FBS. Each electroporation was plated into a 60-mm-diameter tissue culture dish and incubated for 48 h. Forty-eight h after transfection, cells were washed with PBS and lysed using 1×passive lysis buffer, and 20 µL of cell extract was assayed for firefly and Renilla luciferase activity using Dual-Luciferase^®^ reporter Assay System kit (Promega, USA) according to the manufacturer's instructions.

### Western blotting analysis

Whole cell extracts were prepared from cells transiently transfected with SATB1 RNAi plasmids or control plasmids using lysis buffer containing 50 mmol/L Tris, (pH 7.4), 0.5% NP-40 and 0.01% SDS) with a cocktail of protease inhibitors. Total protein (20 µg) was boiled for 5 min in loading buffer, chilled on ice and then separated on sodium dodecyl sulfate (SDS)-polyacrylamide gels. Subsequent to transfer onto PVDF membranes (Bio-Rad, USA), non-specific protein interactions were blocked by incubation in 5% nonfat dry milk in TST buffer (50 mmol/L Tris-HCl, 150 mmol/L NaCl, and 0.05% Tween 20, pH7.6) at 4°C for 1 h. Membranes were then incubated at 4°C overnight with polyclonal anti-SATB1 or anti-actin monoclonal antibody (Sigma, USA) in fresh blocking buffer. Horseradish peroxide-conjugated secondary antibody (R&D, USA) was added for 1 h at room temperature. The blot was developed with ECL reagent (Amersham Biosciences, USA). Prestained markers (NEB, Britiain) were used as internal molecular weight standards.

### RNA isolation and RT-RCR

Total RNA was isolated with Trizol reagent (Invitrogen, USA) according to the manufacturer's protocol. RNA integrity was assessed by visualizing the ribosomal bands on a 1% agarose gel assessed. Finally, cDNA was synthesized from total RNA (1 µg) using AMV Reverse Transcriptase according to the manufacturer's instructions (TOYOBO, Japan), and oligo (dT)_15_ was used as the primer. The reactions were incubated at 42°C for 60 min and then stored at -20°C prior to use. The real-time PCR conditions were 50°C for 2 min, and 95°C for 1 min followed by 40 cycles of denaturation at 95°C for 15 sec, and annealing at 63°C for 1 min.

### Statistical analysis

Results were expressed as mean±SD. Data were analyzed using Student's *t* test. Statistical analysis was performed with statistical analysis software SPSS 10.0. *P* < 0.05 was considered to have statistically significant difference.

## RESULTS

### Identification of SATB1-bound sequences *in vitro* and *in vivo*

To investigate the role of SATB1 in the regulation of the BCL2 transcriptional activity, we first analyzed the region 1.1 kb upstream of the translation start site of the *BCL2* gene, which is ATC-rich, using Genomatix Software (http://www.genomatix.de/index.html). SATB1, as a MBP, prefers sequences that have a characteristic “ATC sequence context”, which is enriched in stretches of DNA sequences containing a mixture of adenine, thymidine and cytosine (but not guanine) on one strand[21]. One SATB1 binding site was identified. The sequence is proximal to the promoter P2, designated as SB1, which is located -217-193 bp upstream of the translational start site.

**Fig. 1:**
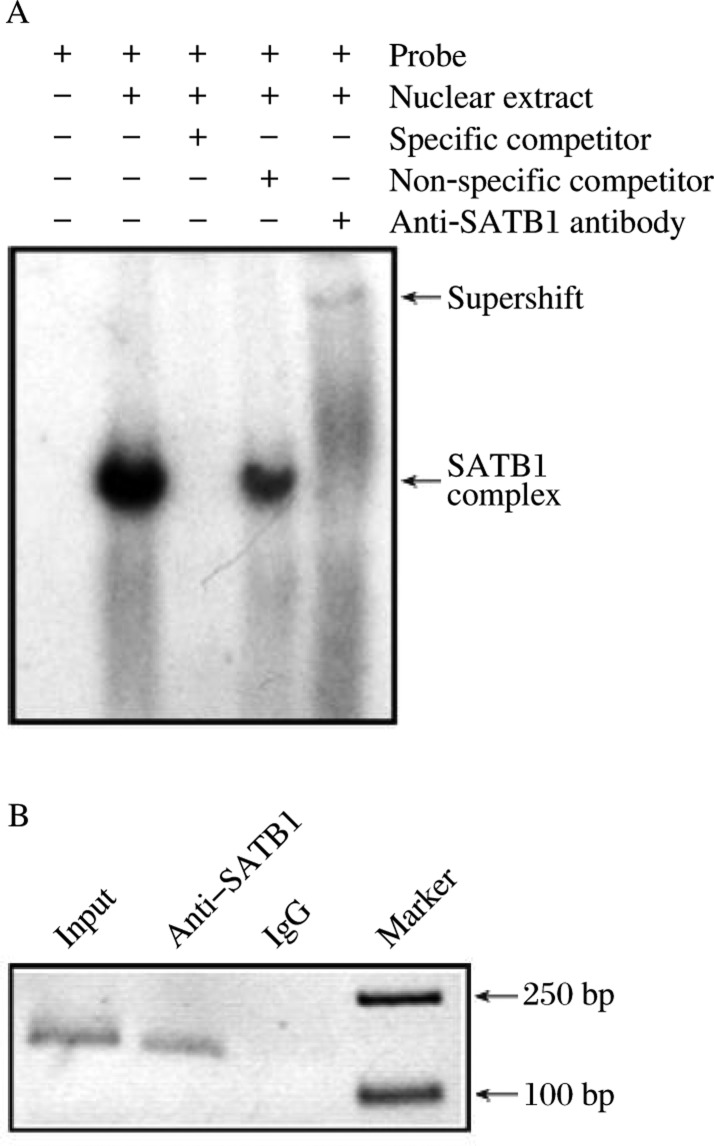
EMSA and ChIP analysis confirm that SATB1 binds to SB1 *in vitro* and *in vivo*. A: EMSA with Jurkat nuclear extracts and probe SB1. Line 1-4 are negative control, positive control, specific cold competitor and non-specific cold competitor, respectively. Line 5 is with anti-SATB1 antibody. The arrows indicate specific DNA-protein complex. B: ChIP analysis of SB1. Precipitated DNA was analyzed by PCR. The PCR products were visualized by ethidium bromide staining of a 1.5% agarose gel.

To confirm the binding of SATB1 to the sequence predicted by bioinformatic analysis, oligonucleotide containing the predicted binding site were radioactively labeled and used as a probe in EMSAs. When the olyonucletides were incubated with nuclear extracts from Jurkat cells, a specific protein complex was formed (***Fig. 1A***). Formation of this complex could be eliminated by a 100-fold molar excess of unlabled probe SB1, but not by 100-fold molar excess of nonspecific olprobe was gonucleotide (***Fig. 1A***). Furthermore, a supershifted complex was detected while anti-SATB1 antibody was present (***Fig. 1A***), suggesting that SATB1 can bind SB1 *in vitro*.

Then we analyzed the *in vivo* SATB1-binding status of SB1 in Jurkat cells by ChIP assay. Chromatin proteins and DNA were cross-linked by formaldehyde treatment in Jurkat cells.The cross-linked chromatin was collected and sheared, and then fractionated using anti-SATB1 antibody as indicated. Negative control is nonspecific IgG. PCR analysis showed that SB1 was specifically immunoprecipitated with anti-SATB1, but not with IgG (***Fig. 1B***). These data demonstrate that SATB1 binds to SB1 in Jurkat cells. Interestingly, SB1 is just located in the region of the negative response element (NRE, -287/-85 bp) of the *BCL2* promoter[6].

### SB1 has negative effect on reporter gene activity

**Fig. 2:**
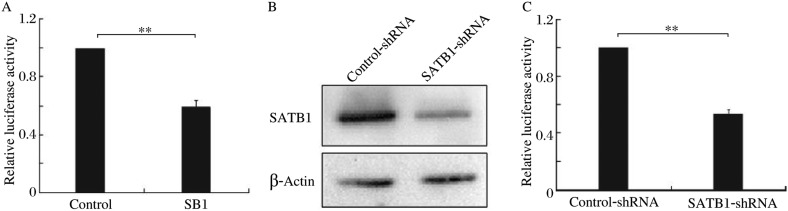
SB1 regulates transcriptional activity in a SATB1-dependant manner. A: Jurkat cells were transiently transfected with SB1 constructs or with the control constructs. The data were normalized relative to the activity of the control luciferase construct that was given a value of 1. B: Western blotting analysis of SATB1 in Jurkat cells transiently transfected with control-shRNA and SATB1-shRNA plasmids, respectively, indicated that SATB1-shRNA effectively reduced SATB1 levels in the cells. C: Cell line that expresses high level of SATB1 (Jurkat) was co-transfected with a construct expressing a short hairpin RNA (shRNA) against SATB1 or unrelated sequence, respectively, together with the SB1 reporter gene constucts. The luciferase activity of the reporter construct with the empty expression vector was defined as 1, and the activity derived from other transfections was normalized to this value (**P < 0.01).

To investigate whether SB1 possesses intrinsic regulatory function, we prepared constructs in which the SB1 sequence was inserted upstream of the luciferase reporter gene under the control of the SV40 promoter. The reporter gene vectors and the control vectors without the SB1 were then transiently transfected into Jurkat cells that were expressing high levels of SATB1, respectively. pRL-SV40 vector was transfected together with the reporter gene as an internal control. We found that SB1 decreased the reporter gene activity to 59% (***Fig. 2A***), suggesting that SB1 is a negative regulatory element.

### SATB1 antagonizes the negative effect of SB1

To evaluate the correlation of SATB1 and the function of the SB1 element, a reporter construct with SB1 inserted upstream of the promoter was cotransfected with SATB1 specific or non-specific siRNA expression plasmids into Jurkat cells that normally express high levels of SATB1 (***Fig. 2B***). As indicated in ***Fig. 2C***, the SB1-reporter gene activity was reduced to 53% when SATB1 was knocked down, which was consistent with our previous study that SATB1 knockdown decreased the expression of BCL2[13]. These data suggest that SATB1 may antagonize the negative effect of SB1 on the transcription of BCL2.

**Fig. 3:**
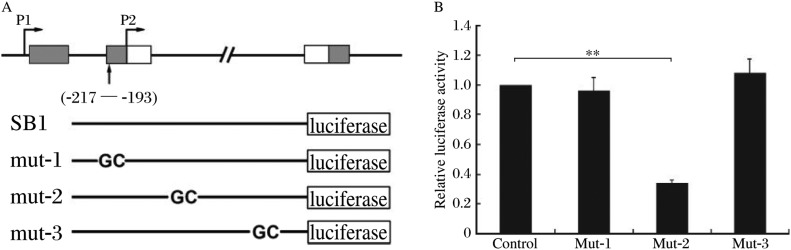
Results of transient transfection with different mutant constructs of SBI. A: A diagram of the *BCL2* gene and the constructs used in the transient transfection assays. All mutant constructs were derived from the -217–-193 construct. B: Results of transient transfection experiments with mutant SB1 constructs or control. The data have been normalized relative to the activity of the control luciferase construct that was given a value of 1. Each experiment was performed at least three times independently and in duplicate. The error bars represent SD. The statistical differences were calculated using student's *t* test (**P < 0.01).

To further confirm the role of SATB1 in the regulation of SB1, reporter constructs containing mutations in SATB1 binding site were generated (***Fig. 3A***). According to the characteristic of the SATB1 binding site, we mutated AT to GC at three sites within the sequence of SB1, respectively. The three constructs containing the first, second or third (5′-3′) mutation sites were named mut-1, mut-2 or mut-3, respectively (***Fig. 3A***). As shown in ***Fig. 3B***, the repression of SB1 on reporter gene was depleted when the first or third AT had been mutated to GC. However, the mut-2 construct repressed the reporter gene activity to 32%, which was more significant than the repression induced by the construct without mutation. These data suggested that the repressive effect of SB1 was mediated by the first and third AT sites cooperatively, while the second AT site was a core for the binding of SATB1, which mediated the antagonizing effect of the protein.

## DISCUSSION

Our study identifies a SATB1 binding site, SB1, located between P1 and P2 region of the *BCL2* gene. It possesses an intrinsic transcriptional regulatory function in Jurkat cells and this function may be related to the transcription factor SATB1.

The region of NRE, which is located between -287 and -85 bp relative to the translation start site of the *BCL2* gene[7], is known not only to suppress the reporter gene activity in Jurkat cells, but also to inhibit expression from the P1 promoter in pre-B cells[6]. The activity of the P1 promoter was higher in the absence of the NRE[14]. Our new identified SATB1 binding site, SB1, is just located within the NRE and can negatively regulate reporter gene activity. Thus, SB1 may contribute to the inhibitory effect of the NRE on P1 activity of the *BCL2* gene. Since P1 is a dominant promoter of the *BCL2* gene in Jurkat cells, we speculate that SB1 is a negative regulatory element that can down-regulate *BCL2* expression in Jurkat cells.

It is known that SATB1 can recruit different transcription factors or chromatin remodeling factors to form protein complexes and regulate a wide variety of genes[22],[25],[26]. The relevance of SB1 regulatory function and SATB1 was thus evaluated with reporter gene system and RNAi experiments. Interestingly, knockdown of SATB1 further enhanced the inhibitory effect of SB1 on the reporter gene activity. It seems that the negative effect of SB1 on transcription activity is independent of SATB1, but can be antagonized by SATB1 binding to SB1.

There is little information regarding the negative regulatory factors binding to the NRE. p53 has been reported to mediate the down-regulation of BCL2 either directly or indirectly through the NRE[7]. However, Jurkat is a wild type p53-deficient cell line[27],[28] and the effect of p53 can be ignored in this cell line. One candidate that may contribute to the negative regulatory function of SB1 within NRE is Oct1, as bioinformatic analysis predicts that the first and third AT sites are both the core sequence of Oct1 binding sites. Oct1 was originally identified as a transcription factor that either positively or negatively regulates gene expression in different tissues[29],[30]. In human T cells, Oct1 has been shown to act as a repressor in concert with YY1 to down-regulate IL-5 and CD21 transcription[31],[32]. It is possible that Oct1 competes with SATB1 to bind to SB1 to regulate the transcription activity. When the expression level of SATB1 is knocked down, Oct1 becomes the predominant regulator and down-regulates the transcription of the *BCL2*.

Additionally, SATB1 may balance the SB1 inhibitory effect caused by negative regulatory proteins through recruiting positive transcription factors to SB1 to form SB1/SATB1 complex. One of the candidate factors recruited by SATB1 to SB1 may be HOX. Our bioinformatic analysis indicates that HOX has binding site that partially overlaps with the SB1 sequence. It belongs to a class of transcription factors called homeobox genes found in clusters named A, B, C and D on four separate chromosomes. Expression of these proteins is spatially and temporally regulated during embryonic development. Among them, HOXA9 is demonstrated to be involved in early T-cell development and apoptosis in primitive thymocytes. Knockout of HOXA9 down-regulates BCL2 expression and delays thymus development in mice[33]. The other candidate is CDX2. Bioinformatic analysis revealed that the 3′ end of SB1 contains a binding site of CDX2. CDX2 is a critical factor for functions of enhancers of different genes[34],[35]. It is also an important factor in mediating the activation of BCL2 in t (14;18) lymphoma cells[8],[36]. It is possible that HOXA9 and CDX2 form a complex with SATB1 at the SB1 site to play a positive role in the regulation of the *BCL2* transcription. Another possibility is that SATB1 may recruit histone acetyltransferases (HATs) or other chromatin remodeling factors to modify the epigenetic status of the promoter region and thus regulate the promoter activity. Confirmation of the candidate proteins binding to SB1 with ChIP or EMSA assays and identification of other unknown components in the SB1/SATB1 complex will provide important clues for understanding the mechanism.

*BCL2* is a proto-oncogene. The critical functions of *BCL2* in apoptosis and the complex structure of the *BCL2* gene provide a very useful model for investigation of transcription regulation. Identification of a new potential negative regulatory element (SB1) within the *BCL2* promoter region may provide an opportunity to enrich our knowledge of gene regulation.
